# FDG-PET/CT of schwannomas arising in the brachial plexus mimicking lymph node metastasis: report of two cases

**DOI:** 10.1186/1477-7819-12-309

**Published:** 2014-10-12

**Authors:** Takaaki Fujii, Reina Yajima, Hiroki Morita, Satoru Yamaguchi, Soichi Tsutsumi, Takayuki Asao, Hiroyuki Kuwano

**Affiliations:** Department of General Surgical Science, Graduate School of Medicine, Gunma University, 3-39-22 Showa-machi, Maebashi, Gunma, 371-8511 Japan; First Department of Surgery, Graduate School of Medicine, Dokkyo University, Tochigi, Japan

**Keywords:** FDG-PET, lymph node metastasis, schwannoma

## Abstract

**Background:**

Schwannoma is a tumor that develops on peripheral nerves or spinal roots. Although any part of the body can be affected, axillar and supraclavicular lesions are unusual for schwannoma. We report two cases of schwannoma arising in the brachial plexus, which were detected by ^18^F-fluorodeoxyglucose positron emission tomography and computed tomography (FDG-PET/CT).

**Case 1:**

A 75-year-old Japanese woman showed high FDG accumulation in a subclavicular or axillary lesion found by FDG-PET/CT. Axillar-subclavicular lymph node metastasis was suspected and surgical excision was performed. Histological evaluation revealed schwannoma.

**Case 2:**

A 75-year-old Japanese woman was diagnosed with suspected primary lung cancer with brain metastases. She showed high FDG uptake at a subclavicular or axillary lesion found by FDG-PET/CT. Surgical excision was performed to arrive at a definitive diagnosis. The mass was located at the trunk of the brachial plexus and was identified as a schwannoma.

**Conclusion:**

Although schwannoma within an axillar or subclavicular lesion is relatively rare, brachial plexus schwannoma should be considered in the diagnosis of masses detected by FDG-PET/CT.

## Background

Schwannoma is a relatively rare neoplasm that arises from Schwann cells of the peripheral nerve sheath [1–3]. Although schwannoma may occur in any organ, including the extremities, trunk and head, it rarely appears as an axillar or supraclavicular lesion.

In recent years, there has been an explosive increase in the use of positron emission tomography (PET) in clinical applications. ^18^F-fluorodeoxyglucose (FDG)-PET is a noninvasive whole-body imaging technique used to evaluate various kinds of malignancy, including colorectal cancer and lung cancer, as well as for tumor staging, restaging, and detection of recurrence, and for monitoring treatment response [4–8]. The FDG-PET technique also has the potential to detect malignant tumors in asymptomatic individuals [9–11]. However, schwannomas generally show high FDG uptake [12–14]. We report here two cases of schwannoma arising in the brachial plexus detected by FDG-PET and initially suspected to be axillar or subclavicular lymph node metastasis.

## Case presentation

### Case 1

**Figure 1:**
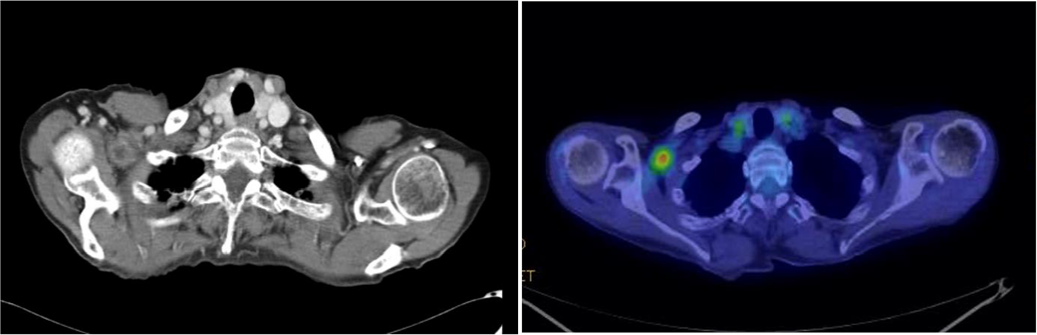
^**18**^ **F-fluorodeoxyglucose positron emission tomography and computed tomography revealed a mass with abnormal uptake of fluorodeoxyglucose in the right axillary to subclavicular region.**

**Figure 2:**
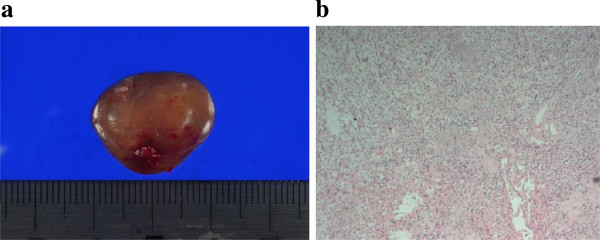
**Histological evaluation. (a)** Macroscopic view. The tumor is an encapsulated mass with a soft and elastic consistency. **(b)** Histological evaluation revealed an encapsulated mass composed of spindle-shaped cells with pointed basophilic nuclei and with nuclear palisading arranged in interlacing bundles known as Verocay bodies.

A 75-year-old Japanese woman was referred to our hospital after high accumulation in a subclavicular or axillary lesion was found by FDG-PET and computed tomography (CT) (SUVmax 3.4) (Figure 1). She had been diagnosed with early rectal cancer and a laparoscopic low anterior resection had been performed 14 months previously. Axillar or subclavicular lymph node metastasis from carcinoma was suspected, but the differential diagnosis of a solid mass with FDG uptake by PET/CT in axillar or subclavicular lesions includes malignant lymphoma, sarcoidosis, tuberculosis, and other infections. For treatment and diagnosis, surgical excision was therefore performed. The tumor was located at the trunk of the brachial plexus, and was identified as a schwannoma. Histological evaluation revealed an encapsulated mass composed of spindle-shaped cells with pointed basophilic nuclei and with nuclear palisading arranged in interlacing bundles known as Verocay bodies (Figure 2). Neither malignancy of the proliferative cells nor invasion was observed. These findings are compatible with schwannoma. Our follow-up of the patient has remained uneventful without postoperative neurological disturbance.

### Case 2

**Figure 3:**
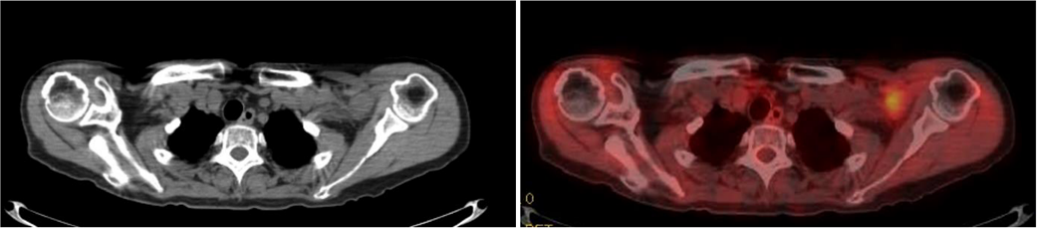
^**18**^ **F-fluorodeoxyglucose positron emission tomography and computed tomography revealed a mass with abnormal uptake of fluorodeoxyglucose in the left axillary to subclavicular region.**

**Figure 4:**
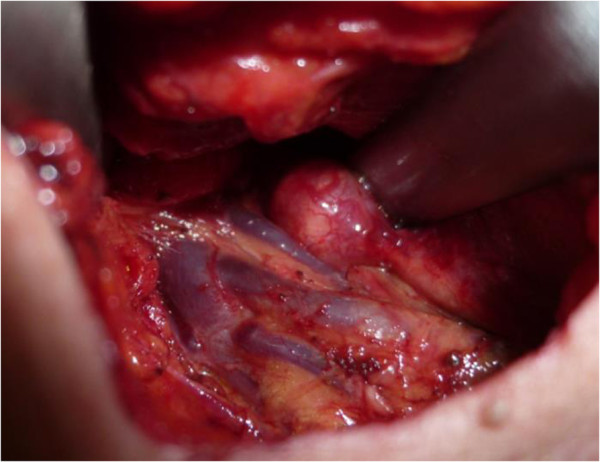
**The mass was located at the trunk of the brachial plexus, and was identified as a schwannoma.**

A 75-year-old Japanese woman showed an abnormal shadow in the right lung and multiple masses in the brain, suggesting primary lung cancer with brain metastases. She showed high FDG uptake in a subclavicular or axillary lesion found by FDG-PET/CT (SUVmax 2.6) (Figure 3), and the SUVmax of the lung masses was 2.7. Thus, axillar or subclavicular lymph node metastasis was suspected and surgical excision was performed to arrive at a definitive diagnosis. The mass was located at the trunk of the brachial plexus, and was identified as a schwannoma (Figure 4). Surgical resection was not performed because injury to the brachial plexus sometimes leads to postoperative neurological disturbance. In this case, because findings at the surgery indicated that the mass was located at the trunk of the brachial plexus, strongly suggesting schwannoma, we did not perform resection. The patient was diagnosed with primary lung cancer by thoracoscopic surgery.

## Discussion

The FDG-PET technique has been widely used with high accuracy for staging and identifying recurrence in various types of cancer [15, 16]. Ueda *et al.* demonstrated that the diagnostic accuracy of FDG-PET/CT for axillary lymph node metastasis in cases with breast cancer was 83%, with 58% sensitivity and 95% specificity [17]. Thus, the false-positive diagnostic rate of FDG-PET/CT appears to be low and FDG-PET is useful for the detection and localization of schwannoma [12–14]. Schwannoma is a tumor that develops on peripheral nerves or spinal roots [1]. The most common locations are the neck, head, extensor surfaces of the extremities, posterior mediastinum, and stomach [2, 3], while axillar and supraclavicular lesions are unusual [16, 18, 19]. The differential diagnosis of a solid mass with FDG uptake by PET/CT in axillar or subclavicular lesions includes lymph node metastasis from carcinoma, malignant lymphoma, sarcoidosis, tuberculosis, and other infections. The distinction of schwannoma in axillary or subclavicular lesions from lymph node metastasis may be difficult. Previous reports have discussed schwannoma cases misdiagnosed as axillary node metastasis in breast cancer patients [18, 19]. In the present cases, PET/CT also failed to distinguish schwannoma from lymph node metastasis. Schwannoma arising in the brachial plexus should be included in the differential diagnosis of masses in the axillar or subclavicular region detected by FDG-PET. Supraclavicular lymph node metastasis is often encountered in patients with lung cancer; however, axillary lymph node metastasis from lung cancer or colon cancer appears to be very rare [20, 21], and the status of the primary lesion should be taken into consideration for the diagnosis.

In Case 2, we did not resect the mass. In cases of schwannoma, resection is performed only if the lesion is symptomatic, moderately large, or exhibits rapid growth. Furthermore, it is necessary to pay close attention to irreversible complications that may arise from resection in the brachial plexus. In this case, because findings during surgery indicated that the mass was located at the trunk of the brachial plexus, strongly suggesting schwannoma, in conjugation with the findings and experience of Case 1, we did not perform resection. When a connection with the brachial plexus is confirmed, resection should be avoided if there are no symptoms.

## Conclusion

We report two cases of schwannoma arising in the brachial plexus and mimicking lymph node metastasis, identified by FDG-PET/CT. Although schwannoma in an axillar or subclavicular lesion is relatively rare, brachial plexus schwannoma should be considered in the diagnosis of masses detected by FDG-PET.

## Consent

Written informed consent was obtained from the patient for the publication of this report and any accompanying images.

## Abbreviations

CT: computed tomography
FDG: ^18^ F-fluorodeoxyglucose
PET: positron emission tomography.
